# Modeling Disordered Regions in Proteins Using Rosetta

**DOI:** 10.1371/journal.pone.0022060

**Published:** 2011-07-29

**Authors:** Ray Yu-Ruei Wang, Yan Han, Kristina Krassovsky, William Sheffler, Michael Tyka, David Baker

**Affiliations:** 1 Graduate Program in Biomolecular Structure and Design, University of Washington, Seattle, Washington, United States of America; 2 Department of Biochemistry, University of Washington, Seattle, Washington, United States of America; 3 Basic Sciences Division, Fred Hutchinson Cancer Research Center, Seattle, Washington, United States of America; 4 Molecular and Cellular Biology Program, University of Washington, Seattle, Washington, United States of America; 5 Howard Hughes Medical Institute, University of Washington, Seattle, Washington, United States of America; University of South Florida, United States of America

## Abstract

Protein structure prediction methods such as Rosetta search for the lowest energy conformation of the polypeptide chain. However, the experimentally observed native state is at a minimum of the free energy, rather than the energy. The neglect of the missing configurational entropy contribution to the free energy can be partially justified by the assumption that the entropies of alternative folded states, while very much less than unfolded states, are not too different from one another, and hence can be to a first approximation neglected when searching for the lowest free energy state. The shortcomings of current structure prediction methods may be due in part to the breakdown of this assumption. Particularly problematic are proteins with significant disordered regions which do not populate single low energy conformations even in the native state. We describe two approaches within the Rosetta structure modeling methodology for treating such regions. The first does not require advance knowledge of the regions likely to be disordered; instead these are identified by minimizing a simple free energy function used previously to model protein folding landscapes and transition states. In this model, residues can be either completely ordered or completely disordered; they are considered disordered if the gain in entropy outweighs the loss of favorable energetic interactions with the rest of the protein chain. The second approach requires identification in advance of the disordered regions either from sequence alone using for example the DISOPRED server or from experimental data such as NMR chemical shifts. During Rosetta structure prediction calculations the disordered regions make only unfavorable repulsive contributions to the total energy. We find that the second approach has greater practical utility and illustrate this with examples from *de novo* structure prediction, NMR structure calculation, and comparative modeling.

## Introduction

Native protein structures frequently have disordered segments which do not adopt single unique conformations in the native state. These include both flexible termini and internal loops. In some cases these segments may adopt unique structures when they interact with binding partners [1], [2], in others, they may remain flexible in all functional states of the protein [3].

Disordered regions present a challenge for structure prediction methods such as Rosetta which treat the prediction problem as a search for the lowest energy state of the polypeptide chain. Such regions do not adopt a single unique conformation, and proper accounting for their contributions to the free energy of the native state requires estimating their entropy as well as energy. This presents two problems: first, computing entropies is difficult and CPU intensive, and second, prediction cannot be treated as a search for the single lowest energy state of the chain.

In this paper we describe two approaches for treating disordered regions in structure prediction calculations. In the first approach, an ensemble of models is generated using standard energy based search methods. For each model, individual regions are allowed to become disordered if the gain in entropy outweighs the loss of attractive energetic interactions. The lowest free energy models are then selected from the population. In the second approach, disorder prediction methods are used to identify in advance regions that are likely to be disordered in the native state. Standard energy based search is then carried out, but the predicted disordered regions are only allowed to make unfavorable steric repulsive interactions with the rest of the chain, which results in folded conformations with widely ranging conformations for the disordered regions which make little overall contribution to the energy. We compare the strengths and weaknesses of the two approaches on a range of protein structure modeling problems.

## Methods

The algorithms and parameter determination for the first class of methods for modeling protein disorder explored in this paper are described in the Results section. Here we focus on the implementation within the Rosetta program of the second approach. In this approach, disordered residues are predicted in advance and then during structure prediction simulations treated as interacting through repulsive interactions only.

### Implementation of the Second Approach

Calculating only repulsive energies at known disordered regions in Rosetta is implemented by creating a new residue type, REPLONLY, and a “mover”, RepulsiveOnlyMover.

Rosetta modeling generally starts with a coarse grained “centroid” representation and then switches over to a higher resolution “all atom” representation. The REPLONLY residue type has both a centroid and all-atom representation. The REPONLY patch replaces all the heavy atoms with a newly defined “REPLS” atom at the centroid stage, and with a “HREPS” atom at the all-atom level. These two artificial new atom types, interact only through repulsive interactions; the vdwscore at centroid level and lennard-jones (LJ) at the all atom level [4]. The atom radius of the new atom types are taken to be the smallest radius of any atom in the atom set (residue) being replaced.

The RepulsiveOnlyMover uses the Mover interface [5] and reads the disordered residue numbers from the command line and replaces those residues with glycines. It then assigns the REPLONLY residue type to those glycines. Because of the glycine replacement, conformations are only penalized if the backbone of the disordered region overlaps with rest of the protein chain; this also reduces computation time at the all atom stage. The REPLONLY assignment prevents disordered residues from interacting favorably with the rest of the protein and with other disordered regions, and hence focuses optimization on the ordered portions of the protein.

To score REPLONLY residues, we modify both centroid and all-atom scoring methods. At centroid level, we retain only the repulsive interactions (the vdw_score). The SecondaryStructuralPotential class identifies and scores secondary structure elements. To turn off those energies, we modified the identify_ss function in SecondaryStructurePotential class to prevent REPLONLY residues being assigned secondary structure which excludes these residus from evaluation of secondary structure packing terms. The remaining centroid level score terms were modified with a simple residue type check prior to energy calculations: If the residue or residue pair (for two-body energies) is designated REPLONLY by the RepulsiveOnlyMover, they are ignored.

At the all-atom level, van der Waals interactions between atoms are explicitly modeled by a LJ potential, which is divided into attractive and repulsive components. The LJ potential is precomputed in a class called Etable, which generates a look-up table by computing pairwise energies in advance (fa_atr/fa_rep from the Lennard-Jones potential and fa_sol from the Lazaridis-Karplus solvation model). The REPLONLY atom types are ignored in the calculation of fa_atr and fa_sol by assigning a value of zero for bins of any pairwise interactions between atoms for which one atom is of type REPLS or HREPS. fa_rep remains unchanged. Since REPLS and HREPS, do not have any hydrogen bond donors or acceptors, they are ignored in the hydrogen bond energy calculations. For the rest of the scoring methods at the all-atom stage, we used a residue type check to skip energy calculations for REPLONLY residues.

## Results and Discussion

### First Approach

#### Free Energy Function

As described in the introduction, our first approach seeks to identify disordered regions from low energy predicted models by optimizing a simple free energy function.

We use a free energy function very similar to that used in previous studies of protein folding mechanisms [6]. Each residue is considered to be either fully ordered or fully disordered. When ordered, the residue has an energy equal to the sum of its interactions with all other ordered residues, but zero entropy. When disordered at the N or C terminus, the residue has entropy *E_d_*/T, but its interaction energies with other residues are all set to zero. The entropy of disordered internal loops is taken to be *β*⋅ln (*L*/*L_0_*))/T, where *L* is the length of the loop. The optimization of *E_d_*, *β* and *L_0_* is described below. Given an assignment of each residue as ordered or disordered, the total free energy, *F*, is then



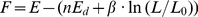
(1)where *E* is the interaction energy for all ordered residues.

Determining the lowest free energy assignment of order/disorder to the structure requires a search over all possible assignments. For computational tractability, no more than two consecutive stretches of disordered regions are allowed. In this case, the lowest free energy assignment can be found by straightforward enumeration. The free energy of the conformation is then taken to be the free energy associated with this optimal assignment. The most likely structure has the lowest free energy, and the extent of order/disorder at a given position is estimated as the frequency with which the residue is ordered in the population of models.

#### Prediction of Disordered Segments at Termini

We began by allowing only disordered N and C terminal segments (referred to as tails throughout the text). Most proteins determined by X-ray crystallography have disordered tails trimmed by crystallographers for easier crystallization or better crystal quality. We spliced tail regions for the gene sequence onto eight proteins where Rosetta makes reasonable predictions (Table 1). In order to study the effects caused by different length of tails, for each of protein in the set, we varied the length of the disordered tail from 2 to 20 amino acids. For each tail length, we then generated a large number of models using the Rosetta *de novo* structure prediction methodology (command lines are provided below).

**Table 1:** Protein sequences used to test the prediction of disordered termini.

PDB code	Constructed N-terminaltail sequence	Core protein sequence	Constructed C-terminaltail sequence
1enh	VYCTRYRRPKQPKDKNTDEK	RPRTAFSSEQLARLKREFNENRYLTERRRQQLSSELGLNEAQ IKIWFQNKRAKI	KKSTGSKNPLALQLMAQGLY
1faa	VVKRKDRRRMRGGEVRASM	LELALGTQEMEAIVGKVTEVNKDTFWPIVKAAGDKPVVLDMF TQWCGPCKAMAPKYEKLAEEYLDVIFLKLDCNQENKTLAKEL GIRVVPTFKILKENSVVGEVTGAKYDKLLEAIQAARS	
1mgw	GPVAAAAPASHAVAASSAAS	ASVKAVGRVCYSALPSQAHDTLDLIDEGGPFPYSQDGVVFQN REGLLPAHSTGYYHEYTVITPGSPTRGARRIITGQQWQEDYY TADHYASFRRVDFAC	
1nps	M	ANITVFYNEDFQGKQVDLPPGNYTRAQLAALGIENNTISSVK VPPGVKAILYQNDGFAGDQIEVVANAEELGPLNNNVSSIRVI SVPV	QPRARFFYKEQFDGKEVDLP
1ten	LHIVKNNTRGPGLKRVTTTR	LDAPSQIEVKDVTDTTALITWFKPLAEIDGIELTYGIKDVPG DRTTIDLTEDENQYSIGNLKPDTEYEVSLISRRGDMSSNPAK ETFTT	GLDAPRNLRRVSQTDNSITL
1b3a	LCAPASASPYSSDTTPCCFA	YIARPLPRAHIKEYFYTSGKCSNPAVVFVTRKNRQVCANPEK KWVREYINSLEMS	
1hz6	PFVENKEETPETPETDSEEE	VTIKANLIFANGSTQTAEFKGTFEKATSEAYAYADTLKKDNG EWTVDVADKGYTLNIKFAG	KEKTPEEPKEEVTIKANLIY
1ctf	SAAAAVAVAAGPVEAAEEKT	EFDVILKAAGANKVAVIKAVRGATGLGLKEAKDLVESAPAAL KEGVSKDDAEALKKALEEAGAEVEVK	

**Figure 1:**
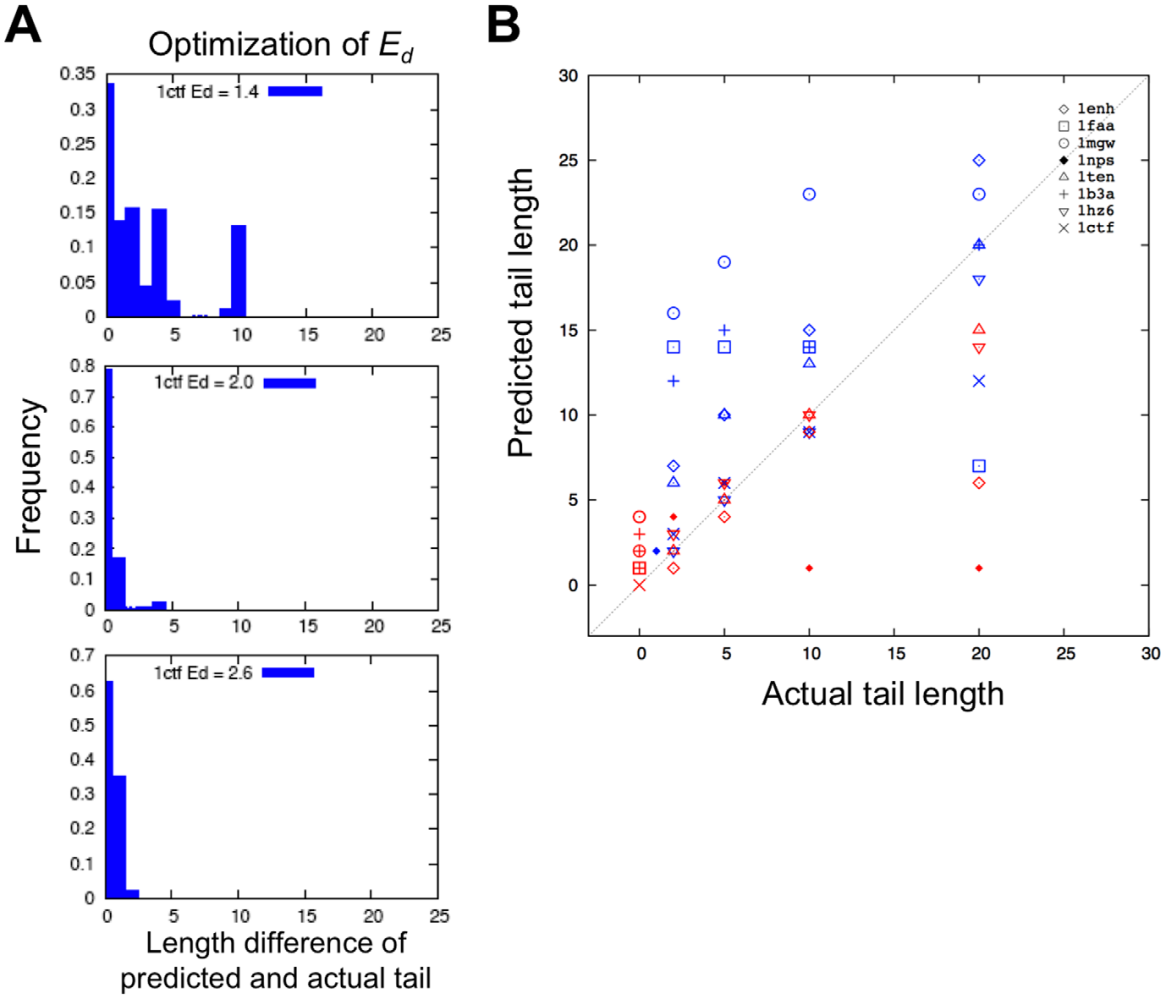
Results of disordered termini prediction. (**A**) Optimization of *E_d_* value using 1ctf from the test set as a representative example. In panel 1 to 3, histograms show the accuracy of prediction results using representative of *E_d_* values, where the x-axis shows the length difference of predicted and actual tail, and the y-axis shows the frequency of prediction. We show here the prediction results with *E_d_* values of 1.4, 2.0 and 2.6 in panel 1, 2 and 3, respectively. With the *E_d_* value of 2.0, the prediction shows the greatest accuracy, where the predicted length difference equals to zero (the prediction matches the actual length) with the highest frequency of 0.8 (maximum equals to 1). (**B**) Prediction of disordered terminal regions. Blue and red symbols represent N- and C- terminal tails, respectively. Different symbols corresponds to different test cases; the multiple instances of each symbol type represent the different tail lengths considered for a given test case.

We used enumeration to identify the lowest free energy state given a set of models (decoys) for a given chain length. For each decoy set, we allowed increasing numbers of residues to be disordered coming in from both the N and C terminus and selected the assignment of disorder which produced the lowest free energy according to Eq. (1). This calculation was carried out for values of *E_d_* between 1.0 and 5.0. Predicted disordered regions were compared to ordered regions observed in crystal structures. A value of 2.0 for *E_d_* gave the most accurate recapitulation of the order observed in the crystal structures (Figure 1 A). This is very close to the value (1.75) used in our earlier models of protein folding kinetics [6]. When restricted to decoys close to the native structure (less than 2 Å C*α*-rmsd), the method is reasonably successful in properly assigning disordered termini (Figure 1 B).

**Figure 2:**
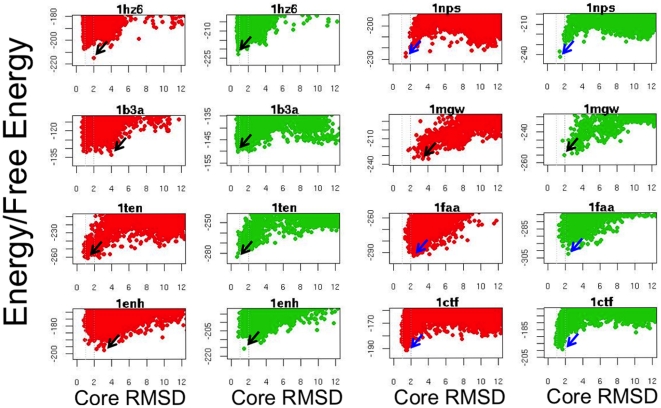
Comparisons of energy versus rmsd (red) and free energy versus rmsd (green) plots for cases with disordered termini but not disordered internal loops. The points in the plots represent Rosetta generated protein structure models. In column 1 and 3, the y axis is the Rosetta all-atom energy, and in columns 2 and 4, the free energy computed using Eq. (1) with structure derived assignment of disorder/order. On the x-axis is the C*α*RMSD deviation (core RMSD) to the folded portion of native structure. Black arrows highlight regions where the free energy landscape provides improved discrimination; blue arrows, where discrimination is equivalent.

As noted earlier, the native structure is at a minimum of the free energy rather than the enthalpy. We compared the minima of the free energy, computed using Eq. (1) with correct assignment of disordered termini, to the minima of the Rosetta energy function. Most of the decoy sets showed equal or better discrimination of decoys with the free energy function, but the differences were not large (Figure 2).

#### Prediction of Disordered Segments at Internal Loops

We tested the method on cases with disordered internal loops using a benchmark set derived from NMR structures (Table 2). For each of 30 amino acid sequences for which NMR ensembles were available, we generated ensembles of Rosetta decoys. We defined residues with mean square deviations in the NMR ensemble of greater than 2Å as disordered; this definition was supported by visual inspection of the ensembles.

**Table 2:** NMR structures used to test the prediction of disordered internal regions.

2jpf	2jqj	2jrk	2jwx	2yw5
2kbg	2kbh	2kbj	2kbk	2kcc
2kcj	2kd5	2kdl	2khc	2kjd
2kjv	2kjw	2kk0	2kmg	2koj
2kpm	2kpn	2kpo	2kpq	2yrz
2kqr	2kre	2krk	2rqp	2rq6

To make the search for the lowest free energy assignment of disorder/order using Eq. (1) tractable, we required that disordered regions be at least 4 residues. At the beginning of the search, we set all of the residues for each of the decoys to be ordered. Then we changed the state of the residues from ordered to disordered, four consecutive residues at a time, and calculated the free energy using Eq. (1). During each round of search, we kept the state that gave the lowest free energy, and went on with next round of search until the free energy could not be lowered any further.

This algorithm was used to assign order/disorder to each residue in each of the decoy structures for each protein in the benchmark set. For each residue in each protein, we calculated the frequency that the residue was predicted to be disordered in the lowest free energy state. We evaluated these predictions by comparing them to the disordered regions identified in the NMR ensembles using a simple scoring scheme



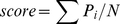
(2)For each residue i, *P_i_* is the frequency in the model calculations of the state (ordered/disordered) observed in the NMR ensemble, and N is the length of the protein. Thus, if all ordered residues are correctly predicted to be ordered, and all disordered residues to be disordered, the score is 1.0.

In our previous work on protein folding pathways, we used values of β and *L_0_* of 1.8 and 0.15, respectively [6]. We optimized these parameters for disordered loop prediction by repeating the search process for different values and determining the score using Eq. (2). Among the values we tried, the results showed no significant preference for particular values for *β* and *L_0_* that satisfy all the proteins in our set; we chose compromise values of 1.5 for *β* and 0.3 for *L_0_*.

**Figure 3:**
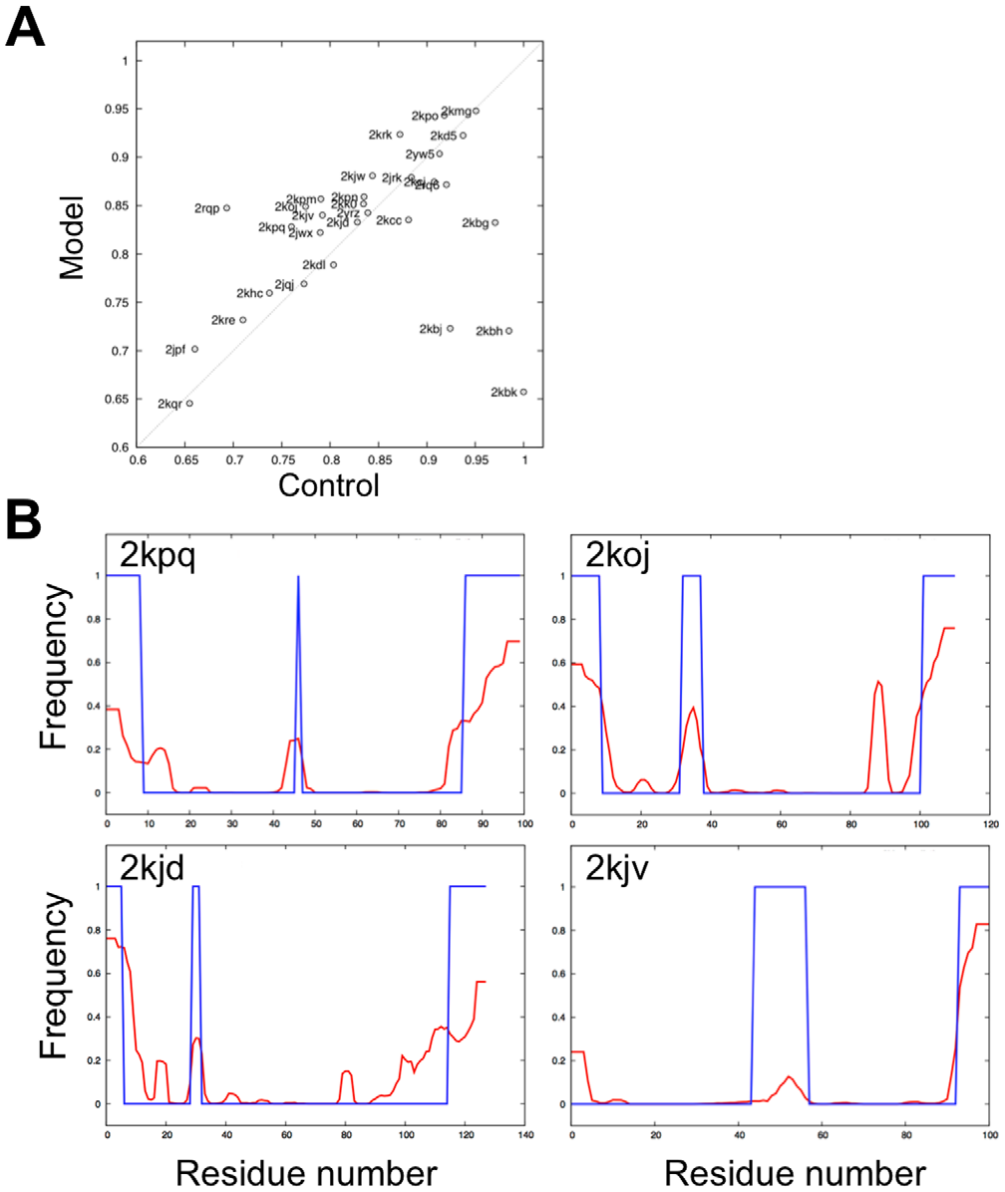
Results of disordered internal loop predictions. (**A**) Comparisons of prediction accuracy using the free energy function with optimized parameters (*β* = 1.5 and *L_0_* = 0.3) with that of a null model. The y-axis shows disorder prediction accuracy over the benchmark set using Eq. (2). The x-axis shows the prediction of the null model, which assumes all residues are ordered. (**B**) Examples of successful prediction of disordered internal loops. Blue line: the actual disordered regions assessed from the residue deviations in the NMR structure. Red line: frequency of disorder assignment by optimization of Eq. (1) over decoy population.

As a control, we compared the prediction accuracy to that of a very simple null model in which all residues are considered to be ordered (this is a reasonable first guess since for the proteins in our test set most residues are ordered). Disorder prediction by minimizing the free energy Eq. (1) results in improvements over the null model in 16 of the 30 test cases, and for 10 cases the predictions are similar or slightly worse (Figure 3 A). Figure 3 B shows examples of good predictions of disordered regions.

**Figure 4:**
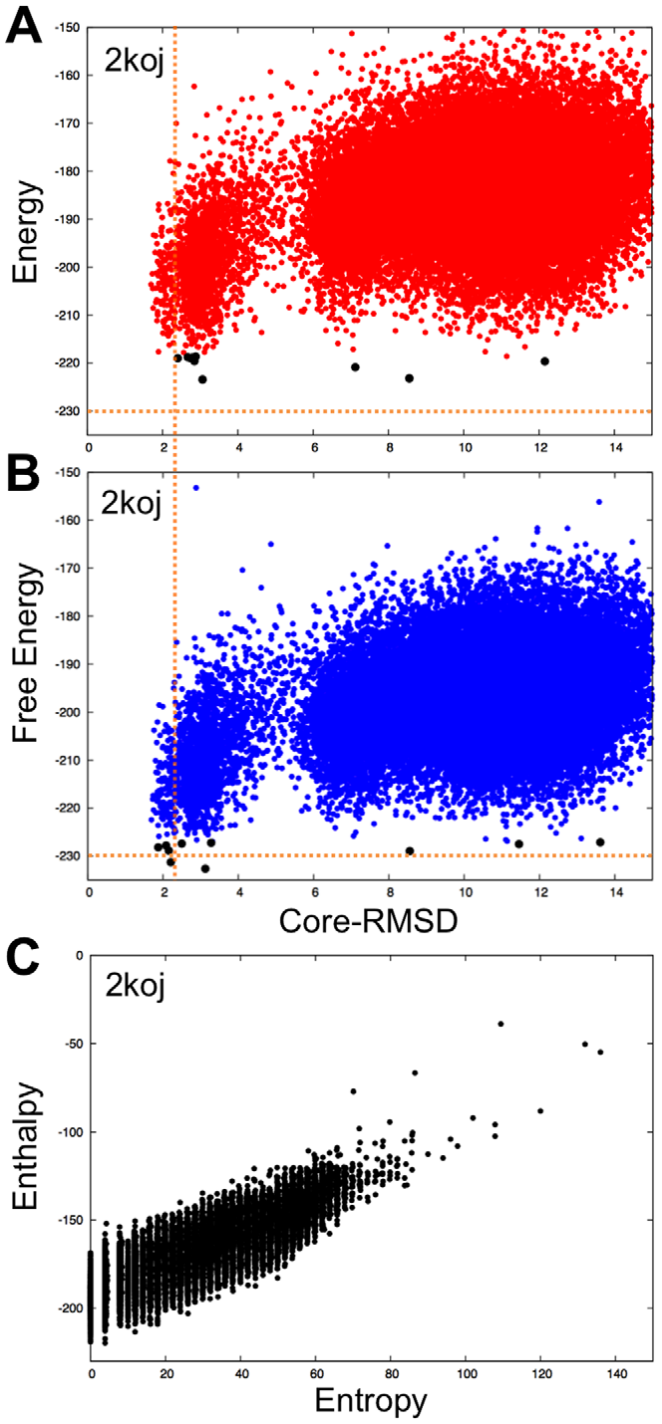
Comparison of energy versus rmsd and free energy versus rmsd plots for case with disordered internal loop (2k0J). A) Rosetta all atom energy and B) free energy computed using Eq. (1) with predicted disordered regions (Fig 3B-2k0j). The energy shown in A is calculated using the Rosetta all-atom energy. In A and B, the x-axis is the RMSD to the folded portion of the native structure. The 10 lowest energy/free energy decoys are shown in black. The dashed orange lines are provided to aid comparison of the two plots. (**C)**. Compensation between the entropic and energetic contributions to the free energy (Eq. (1)).

We next compared the free-energy landscapes computed using Eq. (1) with predicted order/disordered assignments to standard Rosetta energy landscapes for the 30 test proteins. While the free-energy landscapes were consistently displaced vertically relative to the energy landscapes (Figure 4 compare A and B), the overall shapes were very similar. This is a consequence of strong enthalpy/entropy compensation (Figure 4 C); the more residues considered disordered, the more favorable the entropy but the less favorable the energy (enthalpy) as there are fewer interacting residues. Thus, while the free-energy optimization by disorder assignment results in large changes in the energy and entropy, the free energy itself does not change drastically. Such entropy/enthalpy compensation is a ubiquitous feature of physical systems.

### Second Approach

As described above, our first approach involves assignment of disorder to specific regions of the protein chain based on optimizing a simple free-energy function over a set of models generated by Rosetta. Three considerations led us to consider a second approach. First, the method described above depends on the set of Rosetta models used to assign disorder containing some near native structures, which will not always be the case particularly if there are large confounding disordered segments which could hinder search by becoming entangled with the rest of the chain. Second, model discrimination based on the free energy is not very different from discrimination based on the energy due to entropy/enthalpy compensation. Third, modern sequence based disorder prediction methods can be used to reasonably accurately predict disorder before folding calculations are carried out.

Based on these considerations, we developed an approach to utilize sequence based disorder prediction during Rosetta structure prediction calculations. Since these regions can include internal loops, they cannot be simply trimmed from the starting sequence as one would then be left with a disconnected chain. Instead, we chose to model atoms in residues predicted to be disordered as making only repulsive interactions with the rest of the chain. This has the advantage that the chain stays connected, allowing straightforward modeling, but the disordered regions are disfavored from being intercalated into the protein core (no favorable interactions result from this), and residues adjacent to disordered regions must be in positions where there is free space for the disordered segment to fill (ie, they cannot be completely buried). Consistent with intuition, in this approach the energy based model optimization during conformational search, and the subsequent energy based model selection are dominated by the favorable interactions within the ordered region of the protein, with little or no contribution from the disordered segments.

#### Applications of REPLONLY residues in structure calculations

**Figure 5:**
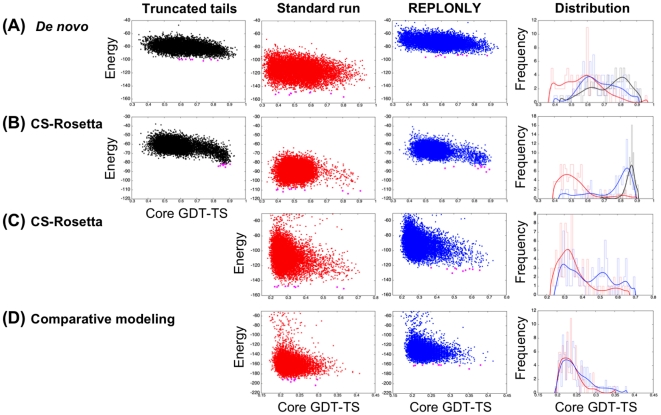
Rosetta modeling with disordered regions treated as REPONLY. (**A**) *ab initio* (1enh). (**B**) CS-Rosetta (2ae9). (**C**) CS-Rosetta (2k4n). (**D**) Comparative modeling (2k4v). Column 1 to 3. Energy *versus* core GDT-TS plots. In column 1 (**Truncated tails**) standard Rosetta simulations are carried out with disordered termini removed, in column 2 (**Standard run**) standard Rosetta simulations are carried out including tails/internal loops and in column 3 (**REPLONLY**) the disordered regions contribute only repulsive-related energies during Rosetta structure calculations. The GDT-TS values on the x-axis were calculated from the folded portion of native structure (Table 4). Column 4: Histograms of core GDT-TS of the 1% low-energy models. Black line “Truncated tails” calculations, red line “Standard run” calculations, and blue line “REPLONLY” calculations.

We tested our second approach on a range of structure modeling problems with both disordered tails and internal loops. We considered three common applications of Rosetta: *de novo* structure prediction, CS-Rosetta structure calculation from NMR chemical shifts, and comparative modeling. In each case, we compared the results with the new method (treating the predicted disordered residues as purely repulsive) to control calculations in which either 1) all residues were considered to be ordered (standard Rosetta calculations) or 2) disordered N or C terminal residues were truncated prior to standard Rosetta calculations (this second control could not be carried out for cases where there were disordered internal loops). The results of the three calculations were compared using the GDT-TS [7] computed over the ordered/structured part of a protein. (Figure 5 A–D and Table 3).

**Table 3:** Modeling with REPLONLY residues.

			10 lowest-energy models		1% lowest-energy models
Prediction methodology	Protein	Run conditions	HighestGDT-TS^a^	Mean (σ^b^) GDT-TS	Mean (σ)GDT-TS
***Ab initio***	1enhA	With tails	0.80	0.55 (0.14)	0.57 (0.12)
		REPLONLY^c^	0.88	0.66 (0.11)	0.67 (0.11)
		Tails trimmed	0.83	0.69 (0.09)	0.73 (0.12)
**CS-Rosetta**	2ae9A	With tails	0.91	0.58 (0.19)	0.53 (0.10)
		REPLONLY	0.90	0.81 (0.09)	0.76 (0.13)
		Tails trimmed	0.91	0.87 (0.02)	0.86 (0.03)
	2k4nA	Typical run	0.62	0.35 (0.14)	0.36 (0.10)
		REPLONLY	0.65	0.53 (0.08)	0.44 (0.13)
**Comparative modeling**	2k4vA	Typical run	0.30	0.24 (0.03)	0.24 (0.03)
		REPLONLY	0.38	0.28 (0.06)	0.25 (0.04)

a GDT-TS was calculated only the folded portion of the native structure.

b σ: Standard deviation.

c Disordered regions were treated as REPLONLY.

#### Ab initio structure prediction

To illustrate the REPONLY method for treating disordered regions in conjunction with traditional Rosetta *de novo* structure prediction, we selected a test case (PDB ID: 1ENH) with disordered 20 residue termini at both ends used in the test of method 1 above (Table 1). We used the DISOPRED2 server [8] to predict the disordered residues, and then assigned those regions as REPLONLY in otherwise standard Rosetta *de novo* folding calculations. Treating the tails as purely repulsive (Figure 5A REPLONLY) yields considerably better models than control Rosetta runs including the termini (Figure 5A. Standard run). The models are similar in quality to those generated in runs in which the termini were trimmed (Figure 5A Truncated tails); the lowest energy models have similar deviations from the native structure (Table 3). While in this case, trimming the disordered regions yielded good results, in other cases we have observed this to yield low energy artifactual structures in which the truncated termini are buried within models leaving no room for the flanking disordered regions (Vernon, Kay, submitted). Truncation also cannot be carried out for internal loops as this would disconnect the chain.

#### CS-Rosetta

CS-Rosetta is a powerful method for determining protein structures from NMR chemical shift data [9], [10]. In this case, rather than using the bioinformatics based DISOPRED method to predict disordered regions, it is more useful to utilize the experimental data, in particular the predicted order parameters (S^2^) [11]. We chose as a cutoff for considering residues to be disordered S^2^ values less than 0.70.

We illustrate CS Rosetta calculations with disordered regions for a case with disordered regions at both termini (PDB ID: 2AE9) and one with a disordered internal loop and relative short disordered termini (PDB ID: 2K4N). For 2AE9, as in the *de novo* modeling case described previously, treatment of the disordered regions as purely repulsive yields results comparable to runs with trimmed tails (Figure 5B–Distribution, compare blue line and black line; Table 3). 2K4N (CASP8 T0460) has a disordered internal loop of 18 residues. During CASP8 we found that in *de novo* folding calculations Rosetta tended to bury this long internal loop region into the core of the protein; we suspected these regions to be disordered from multiple sequence alignments but there was no way to prevent this. Using chemical shifts to pick fragments yielded models with a GDT-TS of 0.62, but as shown in Table 3 and Figure 5C-Standard run, the median GDT-TS of the 10 lowest-energy models is 0.32, which indicates that the low-energy decoys are still dominated by incorrect conformations. Structural inspection suggested that the likely disordered regions were making attractive contacts with residues in the core leading to structural distortion. Treating residues in regions with average S^2^ values less than 0.70 as REPONLY (Table 4) yielded significantly improved results: more high GDT-TS conformations are sampled (Figure 5C-Distribution; compare blue and red) and the low energy decoys have significantly higher median GDT-TS 0.53 (Table 3).

**Table 4:** Test cases for 2^nd^ approach.

Prediction methodology	Disordered regions	Protein	Protein sequence a	Disordered regionsprediction method
***Ab initio***	Terminus	1enhA^b^	VYCTR**YRRPKQPKDKNTDEKRPRTA**FSSEQLARLKREFNENRYLTERRRQQLSSELGLNEAQIKIWFQNKRA**KIKKSTGSKNPLALQLMAQGLY**	DISOPRED2 server^c^
**CS-Rosetta**	Terminus	2ae9A	**MLKNLAKLD**QTEMDKVNVDLAAAGVAFKERYNMPVIAEAVEREQPEHLRSWFRERLIAHRLASV**NLSRLPYEPKLK**	NMR chemical shifts data^d^
	Internal loopplusterminus	2k4nA^e^	**MN**SEVIKEFLEDIGEDYIELENEIHLKPEVFYEVWKYVGEPELKTYVIEDE**IVEPGEYDPPEMKYTNVK**KVKIKKVYFETLDNVRVVTDYSEFQKILKKR**GTKLE**	NMR chemical shifts data^f^
**Comparative modeling**	Internal loops	2k4vA^g^	MFEPGHLHLVS**LPGLDQQ**DINIHIRYEVRQNAESGAYVHFDMDGEIDGKPFSDSFELPRDTAFNFASDATRVAQKHGL**HPKFGAITRVHKEY**DAMFEDIRAKLHAH	Missing density regions

a Residues predicted to be disordered are shown in bold font.

b Assumed from Table 1, tails of 1enh are constructed based on the gene sequence recovered from the gene sequence, in which we assumed these regions likely to be disordered, and was mostly consistent with the prediction results using the DISOPRED2.

c http://bioinf.cs.ucl.ac.uk/disopred/ [8].

d Disordered regions were predicted using “Predicted order parameter (S^2^)” calculated from backbone chemical shifts data with BMRB accession number 6571 [11].

e This is the target T0460 in CASP8 directly downloaded from http://predictioncenter.org/download_area/CASP8/targets/.

f The same method as described on ^d^ with BMRB accession number 15805.

g This is the target T0482 in CASP8 directly downloaded from http://predictioncenter.org/download_area/CASP8/targets/.

#### Comparative modeling

To illustrate the modeling of disordered regions in comparative modeling, we chose the CASP8 target T0482 (2K4V). The template has missing density in several regions; we treated these residues as REPONLY in calculations using the standard Rosetta loop relax protocol using homologue derived constraints (Table 3). Treating the missing density residues as REPONLY yielded improved results over control runs (Figure 5D-Distribution; compare blue and red).

### Command lines

**The command lines used to generate an ensemble of Rosetta decoys in the first approach:**

-frag3 [3mer_fragments_file]

-frag9 [9mer_fragments_file]

-database [path_of_database]

-nstruct 1

-in::file::native [native_pdb]

-rmsd_target [pdb_to_calulate_rmsd_for]

-rmsd_column _trunc

-abinitio::relax

-relax::sequence

-disable_all_filters

-abinitio::increase_cycles 10

-silent_gz

-mute all

-abinitio::rg_reweight 0.5

-abinitio::rsd_wt_helix 0.5

-abinitio::rsd_wt_loop 0.5

-abinitio::use_filters true

-user_tag [tag]

-ex1

-ex2

-steal_3mers

-steal_9mers

-in:file:psipred_ss2 [psipred_file]

**The command lines used for De Novo structure prediction in the second approach:**

-abinitio::increase_cycles 10

-abinitio::relax

-score::weights score13_env_hb

-abinitio::rg_reweight 0.5

-abinitio::rsd_wt_helix 0.5

-abinitio::rsd_wt_loop 0.5

-disable_co_filter true

-frag9 [fragments_file]

-frag3 [fragments_file]

-in::file::fasta [fasta_file]

-in::file::native [native_pdb]

-replonly_residues [residue_numbers_of_disordered_regions]

-correct

-residues:patch_selectors replonly

-mute all

-nstruct 50

### Conclusion

The two approaches we have developed to treat disordered regions in structure prediction calculations differ in both their inputs and their philosphies. The first approach requires no additional input, and seeks to identify likely disordered regions from first principles by explicit optimization of a simple free energy function which balances attractive interactions between ordered regions with increases in entropy accompanying residue disordering. The second approach requires input predictions of likely disordered regions from bioinformatics or experiment, and makes no attempt to model entropic contributions to the free energy. While less elegant, we find the second approach to be more useful in practice as treatment of regions as disordered during structure generation can yield improved models; the first approach can only identify disordered regions following the model generation. We expect the second approach to be useful in a wide range of modeling applications since many biomolecules of interest contain significant disordered regions.
